# Japanese Encephalitis Virus in Meningitis Patients, Japan

**DOI:** 10.3201/eid1103.040285

**Published:** 2005-03

**Authors:** Masaru Kuwayama, Mikako Ito, Shinichi Takao, Yukie Shimazu, Shinji Fukuda, Kazuo Miyazaki, Ichiro Kurane, Tomohiko Takasaki

**Affiliations:** * Hiroshima Prefectural Institute of Health and Environment, Hiroshima, Japan;; † National Institute of infectious Diseases, Tokyo, Japan

**Keywords:** Japanese encephalitis virus, meningitis, PCR, sequence, cerebrospinal fluid

## Abstract

Cerebrospinal fluid specimens from 57 patients diagnosed with meningitis were tested for Japanese encephalitis virus. Total RNA was extracted from the specimens and amplified. Two products had highest homology with Nakayama strain and 2 with Ishikawa strain. Results suggest that Japanese encephalitis virus causes some aseptic meningitis in Japan.

Japanese encephalitis virus is one of the leading causes of epidemic encephalitis worldwide; 35,000–50,000 cases are reported each year with 10,000 deaths (1). Before 1960, >1,000 Japanese encephalitis cases were reported annually in Japan. Because the Japanese encephalitis immunization program was introduced ≈30 years ago, and because of changes in rice farming, the annual number of Japanese encephalitis cases has decreased dramatically. Fewer than 10 Japanese encephalitis cases have been reported annually since 1990 (2). During the past 10 years, only 3 Japanese encephalitis cases have been reported in Hiroshima prefecture, the western part of the main island of Japan (Figure 1); all of these cases were reported in 2002 (2,3).

**Figure 1:**
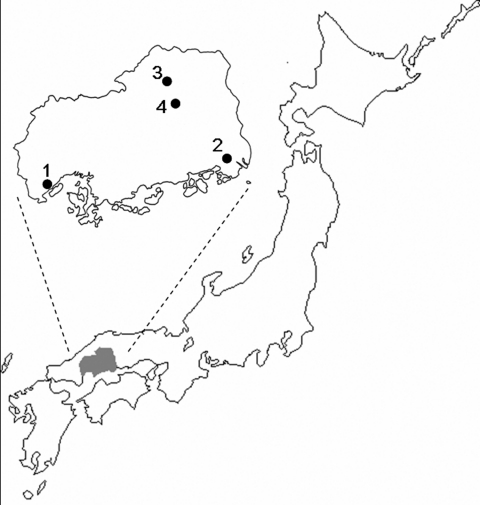
Location of Hiroshima prefecture in Japan and the areas where patients 1–4 resided. Numbers 1–4 correspond to the patients' numbers.

Japanese encephalitis virus causes meningitis as well as encephalitis (4). However, physicians tend not to list Japanese encephalitis virus as a cause of meningitis. In this study, we examined cerebrospinal fluid (CSF) specimens from patients with aseptic meningitis for Japanese encephalitis virus genome.

## The Study

CSF specimens were obtained for diagnostic purposes from 170 patients with a clinical diagnosis of aseptic meningitis from August to October in each year from 1999 to 2002, in Hiroshima prefecture. These CSF specimens were sent to the Division of Microbiology II, Hiroshima Prefectural Institute of Health and Environment, for virologic examination. Viruses were isolated from 112 of 170 CSF specimens in cell cultures by using Vero cells, BGM cells, FL cells, Hep2 cells, and RD18S cells. Enteroviruses and mumps viruses were isolated from 96 and 16 CSF specimens, respectively (Table A1). Thus, etiologic agents were not determined for 58 meningitis cases. CSF specimens from 57 of these 58 cases were used to detect Japanese encephalitis virus genome. Whether the 57 patients had been vaccinated for Japanese encephalitis virus was not known.

Total RNA was extracted from CSF by using ISOGEN-LS (Nippon Gene, Tokyo, Japan). The RNA pellet was resuspended in DEPC Treated Water (Invitrogen Corp., Carlsbad, CA, USA). The RNA was reverse transcribed and amplified by using polymerase chain reaction (PCR) with AMV Reverse Transcriptase XL (Life Sciences Inc., St. Petersburg, Florida, USA.) and Tth DNA Polymerase (Toyobo Co., Ltd., Osaka, Japan) with the primer pair reported by Morita et al. (5). The PCR product was nested PCR-amplified by TaKaRa EX Taq (Takara Bio Inc., Otsu, Japan), with the primer pairs for E gene region reported by Kimura et al. (6); JEN_ATC GTG GTT GGG AGG GGA GA(1147-1166)_JENR_AGC ACA CCT CCT GTG GCT AA(1472-1453). The DNA amplicons were separated by electrophoresis on 1.5% (wt/vol) agarose gel, followed by staining with ethidium bromide (1 µg/mL). The target band detected by electrophoresis was purified by using the Qiagen gel extraction kit (QIAGEN Inc., Valencia, CA, USA). Purified cDNA was directly sequenced in both directions by using the sense and the antisense primers JEN and JENR. The samples that could not be sequenced using these primers were reverse transcribed and PCR-amplified, nested PCR-amplified, and directly sequenced by using other primers designed to amplify the Japanese encephalitis virus E gene region; RT-PCR JEen37s-first: AAG GAG CCA GTG GAG CCA CTT, JEen329c-first: TTC CCG AAA AGT CCA CAT CC, nested PCR and sequence JEen98s-second: CAT GGC AAA CGA CAA ACC AAC, JEen301c-second: CAG TRA AGC CTT GTT TGC ACA C. The samples that could not be directly sequenced by using the second primer sets were amplified with nested PCR and sequenced by using JEen98s-second and JEen271r-inner (RGT RAA GCC TTG TTT GCA CAC).

**Figure 2:**
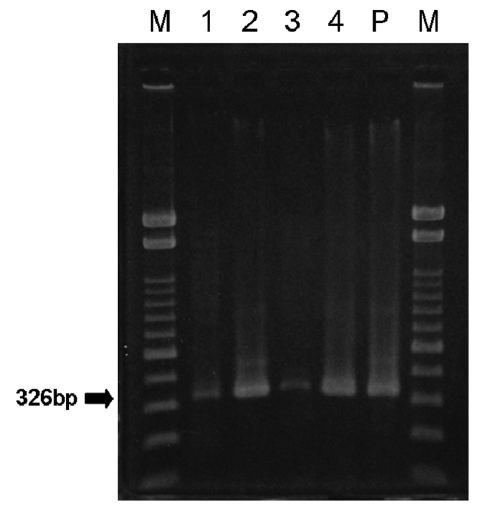
Detection of the Japanese encephalitis virus E gene in cerebrospinal fluid from patients with meningitis. Lanes 1–4, patients Numbers 1–4; lane P, positive control (JaGAr#01); lane M, marker (100-bp ladder).

Electrophoresis demonstrated the band with expected size of 326 bp in 4 of 57 PCR products (Figure 2). Two (numbers 1 and 2) of these products were sequenced on 326 bp and 247 bp, respectively, and the highest homology was with Japanese encephalitis virus, Nakayama strain (genotype III). PCR products 3 and 4 were sequenced on 121 bp and 187 bp by using other primers, and the highest homology was with Japanese encephalitis virus, Ishikawa strain (genotype I) (Table 1).

**Table 1:** Comparisons of the nucleotide sequences of Japanese encephalitis virus E gene detected in cerebrospinal fluid (CSF) with reference Japanese encephalitis virus strains

Patient number and length of sequence	Identity of nucleotide (%)				
	Ishikawa* Genotype I (Ref. 5)	Shizuoka33* Genotype I (Ref. 6)	JaGAr#01* Genotype III(8) (Ref. 7)	Nakayama* Genotype III(8) (Ref. 8)	JKT7003* Genotype IV(9) (Ref. 9)
1 (326 bp)	87.5	88.7	95.4	96.3	80.4
2 (247 bp)	86.2	87.4	96.0	96.4	81.3
3 (21 bp)	98.3	97.5	89.3	89.3	90.1
4 (187 bp)	99.5	98.9	89.8	89.8	87.7

*GenBank accession numbers of these strains are AB051292, AB112703, U44964, U44966, and U70408.

Patients 1–4 lived in Hiroshima prefecture (Figure 1). Patient 1 was a 3-year-old boy and patient 4 was a 6-year-old girl (Table 2). They became sick in early August 2000. Patient 2 was a 2-year-old girl who became sick in late August 2000. Patient 3 was a 4-year-old boy who got sick in mid-September 2000. CSF samples were obtained 2–3 days after the onset of illness. All 4 patients had symptoms characteristic of meningitis and were clinically diagnosed with meningitis.

**Table 2:** Summary of the meningitis patients from whom Japanese encephalitis virus genome was detected by reverse transcription–polymerase chain reaction

Number	Age (y)	Sex	Date of onset	Sampling date	Fever (°C)
1	3	Male	Aug. 5, 2000	Aug. 7, 2000	40
2	2	Female	Aug. 27, 2000	Aug. 7, 2000	Unknown
3	4	Male	Sep. 14, 2000	Sep. 14, 2000	39.5
4	6	Female	Aug. 4, 2000	Aug. 7, 2000	37

## Conclusions

Enteroviruses are the most frequent cause of aseptic meningitis in summer and autumn in Japan (Table A1). In the CSF samples from these 4 patients, however, enteroviruses were not found. Watt et al. reported that in Thailand, 14% of adult patients with acute, undifferentiated fever had a diagnosis of Japanese encephalitis virus infection because anti–Japanese encephalitis virus immunoglobulin (Ig) M antibodies were found (10). Many physicians in Japan consider Japanese encephalitis virus to be mainly associated with encephalitis, and examination for the virus is not usually conducted for patients with meningitis or undifferentiated fevers.

Three Japanese encephalitis cases occurred in 2002 for the first time in 12 years, but anti–Japanese encephalitis virus IgM antibodies were detected in porcine serum samples every year in Hiroshima prefecture (data not shown). So we expected that Japanese encephalitis virus might be associated with aseptic meningitis, and CSF specimens from 57 cases during the past 5 years (1998–2002) had been stored at –30°C in Hiroshima Prefectural Institute of Health and Environment. Those specimens were used to detect Japanese encephalitis virus genome retrospectively. Four of 57 CSF samples were Japanese encephalitis virus genome–positive by nested PCR. We may find more febrile or meningitis cases caused by Japanese encephalitis virus if we routinely conduct serologic or molecular diagnostic tests for Japanese encephalitis virus. CSF samples were from infants and children. Most Japanese encephalitis patients in Japan are >55 years of age. Japanese encephalitis meningitis may also occur among elderly meningitis patients. The Japanese encephalitis vaccination coverage of children has been decreasing in Japan recently; <10 Japanese encephalitis cases are reported annually. If the general population recognizes that Japanese encephalitis virus causes aseptic meningitis at a higher rate than expected, the percentage of Japanese encephalitis vaccination may increase, and the emergence of Japanese encephalitis will be prevented. In 2000, anti–Japanese encephalitis virus IgM antibody was first detected in porcine serum samples in late July, in Hiroshima prefecture (data not shown). This finding suggests that Japanese encephalitis virus was active when these 4 meningitis cases occurred. Further, we found evidence that Japanese encephalitis viruses belonging to genotypes I and III were active in Hiroshima prefecture (data not shown). The results of this study suggest that Japanese encephalitis virus should be considered in the differential diagnosis of aseptic meningitis in areas where Japanese encephalitis is endemic or epidemic.

## Appendix

**Table A1:** Virus isolation or detection by polymerase chain reaction (PCR) in cerebrospinal fluid samples obtained from meningitis patients

Year	No. of cases	Virus isolation or detection by PCR	
		Negative	Positive (detected viruses)
1999	5	2	3 (3 enteroviruses)
2000	37	24	13 (7 enteroviruses, 6 mumps virus)
2001	32	17	15 (9 enteroviruses, 6 mumps virus)
2002	96	15	81 (77 enteroviruses, 4 mumps virus)
Total	170	58	112 (96 enteroviruses, 16 mumps virus)
